# Evaluation of four instruments with different working motion using artificial plastic model with C-shaped single canal

**DOI:** 10.1007/s10266-018-0348-3

**Published:** 2018-02-10

**Authors:** Miki Sekiya, Munehiro Maeda, Ichiroh Katsuumi, Masaru Igarashi

**Affiliations:** Department of Endodontics, The Nippon Dental University School of Life Dentistry at Tokyo, 1-9-20 Fujimi, Chiyoda-ku, Tokyo, 102-8159 Japan

**Keywords:** C-shaped root canal, Canal model block, Stainless steel instruments, Nickel–titanium rotary instruments, Micro-CT

## Abstract

The purpose of this study was to evaluate four instruments with different working motion for preparation of a C-shaped single canal wall using the same artificial plastic models reproduced from a human tooth. One tooth with root canal morphology C1 (the shape is an uninterrupted “C” with no separation or division) was selected among three-dimensional micro-computed tomography (micro-CT) imaging data of extracted human teeth. Imaging data were then converted into STL form data, and twenty-four C-shaped root canal model blocks were manufactured using this STL form data. These blocks were randomly divided into four groups of six blocks each and instrumented as follows: stainless steel K-files (SSK), Self-Adjusting File (SAF), ProTaper NEXT (PTN) and RECIPROC (REC). Micro-CT images taken before and after canal preparation were superimposed, and instrumented canal area, percentage of instrumented canal area, part of instrumented canal area, volume of instrumented canal and time taken for instrumentation were evaluated for each group. The greatest instrumented canal area, percentage of instrumented canal area and volume of instrumented canal were as follows (in decreasing order): SSK > SAF > PTN > REC (*P* < 0.05). The longest time taken for instrumentation was as follows (in decreasing order): SAF > SSK > PTN > REC (*P* < 0.05). The conscious shaping of SSK and the lattice structure of SAF were instrumented all root canal walls equally. PTN and REC required less time taken for instrumentation, but showed unequal instrumentation.

## Introduction

The C-shaped canal is an anatomic feature in which the mesiobuccal root and distal root of the mandibular molar are fused with a semi-circular slit. This structure was first described by Cooke and Cox [1] in 1979; since then, this unique feature and applicable root canal treatment methods have been studied by many researchers [2–12]. Its incidence varies according to ethnicity and is higher in Asian populations when compared with other populations. In particular, the incidence of C-shaped canals in Japanese [13], Chinese [14] and Koreans [15] is > 30%. C-shaped canals are mostly seen in the mandibular second molar, but have also been reported in maxillary molars, the mandibular first molar and the first premolar; thus, it may often be encountered in clinical practice [16–18]. In addition, the C-shaped root canal system varies in form along the root depth [5, 8]. These complex root canal systems from the root canal orifice to the apex are prone to inadequate endodontic treatment, transportation and perforation. However, diagnosis is difficult with traditional two-dimensional radiography. For an accurate diagnosis, various radiographic methods and three-dimensional (3D) cone-beam computed tomography are necessary. These factors make cleaning, shaping and obturation difficult. Thus, endodontic treatment of teeth with C-shaped root canals remains challenging.

Nickel–titanium (Ni–Ti) rotary instruments used in rotary motion are effective for simple root canal systems; however, they have limited use in complex systems, including C-shaped root canals. Although many studies have evaluated root canal preparation for C-shaped canals, most used extracted human teeth that did not contain completely standardized root canal systems [19–21]. Furthermore, some studies have reported that coronal access preparations affect evaluations after canal preparation [19]. Based on these reports, this study was designed to manufacture identical root canal model blocks that reproduced the C-shaped root canal system of the human tooth for evaluation of endodontic treatment. In addition, the present study aimed to more accurately evaluate root canal preparations by omitting access cavity preparation and standardizing measurement areas.

The purpose of this study was to evaluate four instruments with different working motion for preparing the C-shaped single canal wall using the same artificial plastic model reproduced from STL form data of an extracted human tooth. The following parameters were investigated: instrumented canal area, percentage of instrumented canal area, part of instrumented canal area, volume of instrumented canal and time taken for instrumentation.

## Materials and methods

### Manufacture of C-shaped root canal model blocks

**Fig. 1:**
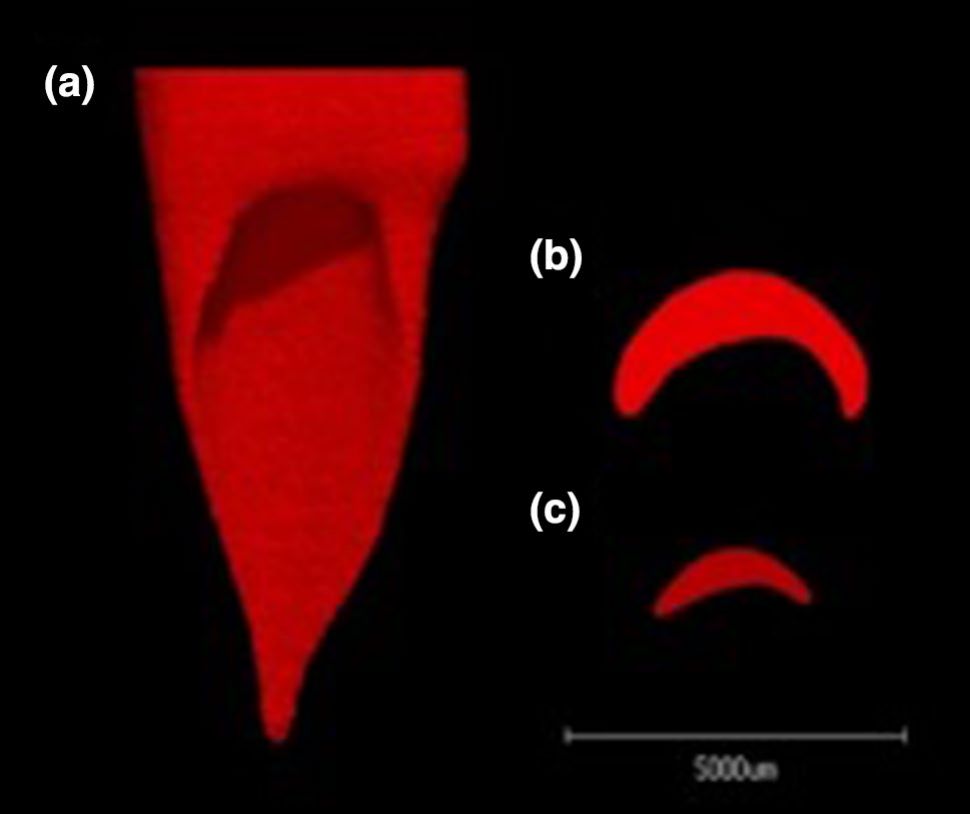
The morphology of the root canal model block. The diameter of apical foramen is 0.17 mm or less and the root canal length is 10 mm. **a** from lingual side; **b** cross sections at 6 mm from the apical foramen; **c** at 3 mm from the apical foramen

Eleven mandibular molars with fused roots were selected from Japanese teeth extracted due to severe periodontal disease and stored in 10% neutral buffered formalin solution. The teeth had full coronal and root anatomy. After washing and drying, teeth were scanned by micro-computed tomography (micro-CT; ELE-SCAN, Nittetsu-Elex, Tokyo, Japan) under the following settings: 52.9 μm at 80 kV and 60 μA. 3D structuring was carried out by image processing software (TRI/3D-Bon, Ratoc System Engineering Co., Tokyo, Japan) and one tooth having root canal morphology C1 (the shape is an uninterrupted “C” with no separation or division) according to anatomical classification by Fan et al. [8] was selected. The 3D image of the selected tooth was converted into STL form data. The same shaped twenty-four canal model blocks were designed based on STL form data, and were manufactured from epoxy resin (S4-SC.98; Nissin Co., Kyoto, Japan). These C-shaped root canal model blocks reproduced the root canal system from the cementoenamel junction to the root apex (Fig. 1). This study was approved by the Ethics Committee of Nippon Dental University (NDU-T2015-33).

### Preparation of C-shaped root canal model blocks

Each block was scanned by micro-CT (ScanXmate-D100SS270; Comscantecno, Kanagawa, Japan) before canal preparation under the following settings: 23.7 µm at 65 kV and 100 µA. During scanning, stage position conditions were standardized. Blocks were scanned at the same position using a pedestal made from silicone rubber impression material and a plastic container. Scanned blocks were confirmed to contain a glide path with the #15 21 mm K-file (MANI, Tochigi, Japan) and a working length of 9 mm was established. The circumference of the blocks was covered by opaque tape to mask the root canal system, and blocks were then randomly divided into four groups of six blocks each and instrumented as follows: stainless steel K-files (SSK), Self-Adjusting File (SAF; ReDent Nova, Ra’anana, Israel), ProTaper NEXT (PTN; Dentsply Maillefer, Ballaigues, Switzerland) and RECIPROC (REC; VDW, Munich, Germany). Based on Paqué et al. [22], the apical preparation was to a size #25. Canals were always filled with distilled water and all canal preparations were completed by one operator following the manufacturers’ instructions.

### SSK Group

#15, 20 and 25 21 mm K-files were used to the working length, and #30, 35 and 40 21 mm K-files were used in a step-back technique along the external form of the root canal orifice. The main preparation method was based on a circumferential filing motion. Tiny-turn and pull motion was added for curved and narrowed areas that showed resistance during circumference filing. Frequent, copious irrigation with distilled water using Terumo Syringe 2.5 ml (Medical Co., Tokyo, Japan) and root clean needle 25G (NISHIKA, Shimonoseki, Japan), and frequent recapitulation with the #15 21 mm K-file to the working length were performed after each instruments.

### SAF Group

The glide path was further prepared using the #20 21 mm K-file. The SAF (*φ* = 1.5, 21 mm) was inserted into the canal along the glide path from one of the mesiodistal ends of the C-shaped root canal to the working length. The SAF was operated in the canal for 4 min using an Osada HL-C handpiece (Osada, Tokyo, Japan) with a light pecking motion. This operation was carried out at 4000 vibrations/min of vertical vibration with amplitude of 0.4 mm. During canal preparation, continuous irrigation was performed with distilled water connected to the SAF irrigation barb by an irrigation tube. After instrumentation was completed on one side, the same preparation method was performed on the opposite side.

### PTN Group

The X1 file (#017/0.04, 21 mm) was passively inserted into the canal along the glide path from one of the mesiodistal ends of the C-shaped root canal to the working length using a brushing motion. When the working length was reached, the X1 file was pulled out and directed against the opposite side. The X2 file (#025/0.06, 21 mm) was used similarly to the X1 file. All files were attached to the X-Smart Plus endodontic motor (Dentsply Maillefer) using the ProTaper NEXT mode (rotary speed, 300 rpm; torque, 2.0 N cm) of clockwise continuous rotary file system. Frequent, copious irrigation with distilled water, debris removal from the flutes and frequent recapitulation with the #15 21 mm K-file to the working length were frequently performed after each instruments.

### REC Group

The R25 file (#025/0.08, 21 mm) was attached to the X-Smart Plus endodontic motor using the RECIPROC mode of reciprocating system. The R25 file was passively inserted into one of the mesiodistal ends of the C-shaped root canal along the glide path. One in-and-out pecking motion with an amplitude of 3 mm using light apical pressure was considered to be one peck; three pecks were performed. Frequent, copious irrigation with distilled water, debris removal from the flutes and frequent recapitulation with the #15 21 mm K-file to the working length were performed each after three pecks. This process was considered to be one cycle and three cycles were repeated until 1 mm short of the working length. As soon as the working length had been reached once, the R25 file was removed from the canal and the same preparation method was performed from the opposite side.

### Evaluation of root canal preparations

After canal preparation, each block was scanned by micro-CT and the obtained images were superimposed on images taken before canal preparation with the TRI/3D-ADJ optional feature (Ratoc System Engineering Co.). Measurements were taken from the root apex to the floor of the pulp chamber (8 mm). Using superimposed images, the instrumented canal area and percentage of instrumented canal area were measured in each group. In addition, part of the instrumented canal area was analyzed. Volume of the canal before and after canal preparation was calculated and the difference was considered to be the volume of instrumented canal. Finally, the time taken for instrumentation was recorded. For SSK, PTN and REC, recording started after confirmation of the glide path with the #15 K-file. For SAF, recording started after establishment of the glide path with the #20 K-file and when the SAF could be freely inserted to the working length. Times for irrigation and exchange of instruments were excluded from the measurement time.

### Statistical analysis

Data are shown as mean ± standard deviation (SD). All statistical analyses were performed using SPSS Statistics version 25 (IBM Japan, Tokyo, Japan). After conducting the Shapiro-Wilk test to evaluate normality, one-way analysis of variance was used to assess equality of variances. Tukey’s test was used when variances were homogeneous and Games-Howell test was used when variances were heterogeneous. Values of *P* < 0.05 were considered statistically significant. *P < 0.05; **P < 0.01.

## Results

**Fig. 2:**
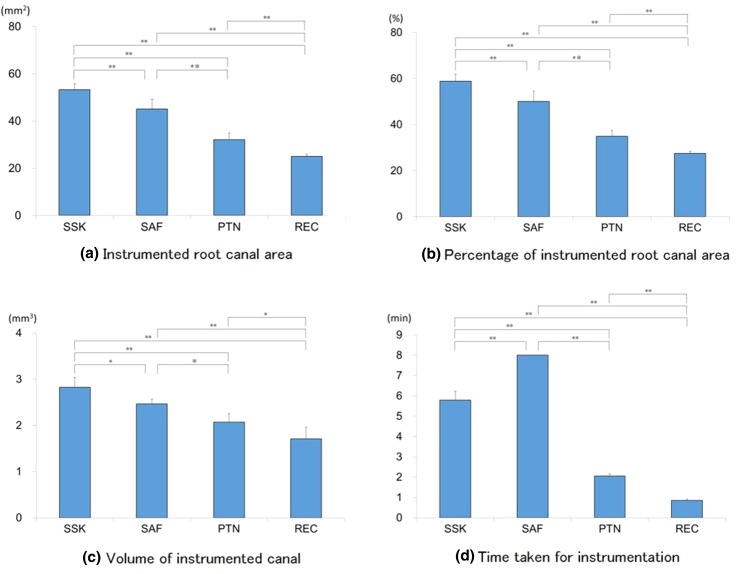
Comparison of four instruments by each parameter

The value of each parameter based on superimposed images is shown in Table 1. In addition, comparisons of each instrument are shown in Fig. 2.

**Table 1:** Values of each parameter for root canal preparation by superimposed images (mean ± SD)

Group	Area		volume (mm^3^)	Time (min)
	Area (mm^2^)	Percent (%)		
*Instrumented root canal*				
SSK	53.20 ± 2.53	58.79 ± 3.09	2.82 ± 0.21	5.78 ± 0.43
SAF	45.12 ± 4.06	50.02 ± 4.58	2.46 ± 0.11	8.00 ± 0.00
PTN	32.16 ± 2.80	34.86 ± 2.77	2.07 ± 0.18	2.06 ± 0.11
REC	25.02 ± 0.90	27.47 ± 1.03	1.71 ± 0.25	0.86 ± 0.06

Statistically significant differences were found between all groups of each parameter (*P* < 0.05)

### Instrumented canal area

The greatest value for each group was as follows (in decreasing order): SSK > SAF > PTN > REC. Differences in areas were statistically significant between all groups (*P* < 0.05, Tukey’s test).

### Percentage of instrumented canal area

The greatest value for each group was as follows (in decreasing order): SSK > SAF > PTN > REC. After angular transformation, these differences were statistically significant (*P* < 0.05, Tukey’s test).

### Part of instrumented canal area

**Fig. 3:**
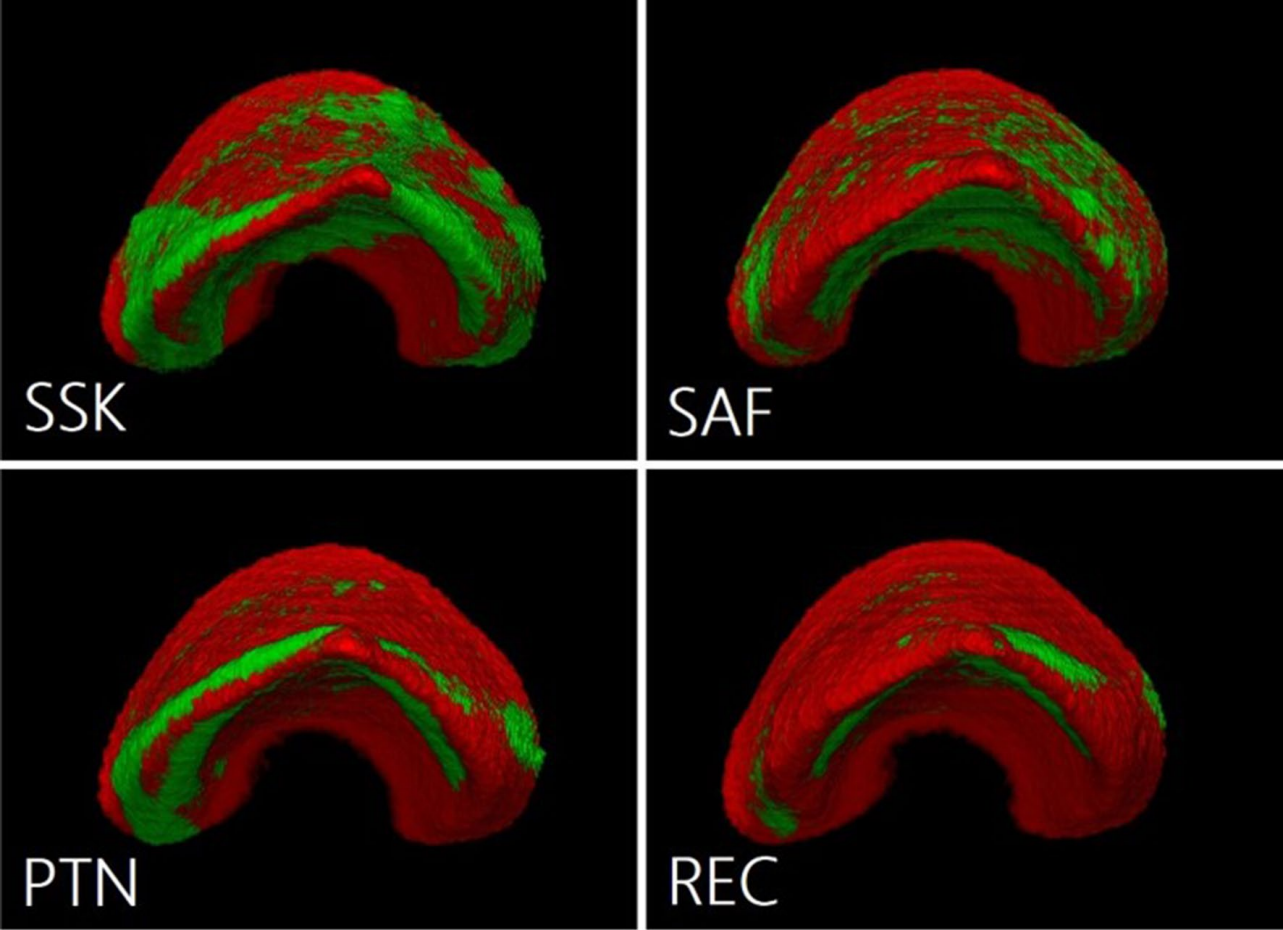
Superimposed images from root apex side for each group. Red represents the form before canal preparation. Green indicates the areas affected by canal preparation

Superimposed images from the root apex side in each group are shown in Fig. 3. SSK and SAF instrumented all root canal walls equally. In contrast, PTN and REC selectively instrumented only the file insertion area.

### Volume of instrumented canal

The greatest value for each group was as follows (in decreasing order): SSK > SAF > PTN > REC. Differences in volumes were significant between all groups (*P* < 0.05, Tukey’s test).

### Time taken for instrumentation

The longest time was as follows (in decreasing order): SAF > SSK > PTN > REC. Differences in amount of time taken were significant between all groups (*P* < 0.05, Games-Howell test).

## Discussion

The recent application of 3D technology has been remarkable in the field of dentistry [23, 24]. To date, many studies have reported the development, improvement and usefulness of root canal models for endodontic education and study [25–28]. However, few models have reproduced the complex root canal system seen in human teeth. The present study used the same shaped artificial plastic model reproduced from the STL form data of an extracted human tooth, which enabled more accurate evaluation of root canal preparations.

In this study, four instruments with different working motion were used for evaluation of a C-shaped single canal wall. SSK is the first choice of root canal instrument due to high versatility. The Hedström file (H-file), the same stainless steel file, is much more efficient than SSK when considering cutting efficiency. However, in clinical practice, the H-file cuts the root canal wall too much because of the high cutting efficiency. A thin root canal wall caused by excessive cutting may lead to root fracture. In addition, the H-file tends to cause inadequate instrumentation such as ledge and strip perforation due to straightening of the root canal. Therefore, SSK was used in this study as the most standard method in endodontic treatment. In hand file technique, root canal walls can be selectively instrumented by altering the preparation and holding methods of the dentist. With regard to SSK, circumferential filing as the main preparation method and filing sensation during the tiny-turn and pull motion for curved and narrowed areas, which showed resistance at the time of circumferential filing, were thought to produce an irregularity of the shaping area. However, root canal preparation by hand files is time-consuming and labor-intensive.

Conversely, Ni–Ti alloy has a shape memory effect and superelasticity. Recently, the use of Ni–Ti rotary instruments has become common because they can rapidly shape root canal walls using a tapered design file and mechanical root canal preparation with a low-speed rotary engine. However, Ni–Ti rotary instruments fracture easily due to low torque at fracture values. Therefore, PTN and REC, which have greater resistance to cyclic fatigue and flexibility by M-wire, have been developed to prevent the fracture of Ni–Ti rotary instruments [29–31]. These systems perform instrumentation with fewer files, using the dedicated engine which rotary speed and torque value were set up. PTN completes the basic instrumentation process with two instruments. PTN, has a variable percentage tapered design and an offset cross section design, reduced the load on the cutting part and limited debris out of the canal by further minimizing the engagement between the file and dentin. Furthermore, REC is the simple and epoch-making system with one instrument, which was designed for use in the reciprocating movement. Nonetheless, root canal preparation with Ni–Ti rotary instruments is poor for complex root canal systems such as oval and C-shaped root canals [19]. The cross section of a non-circular root canal contains an excessively instrumented area, which is shaped by the circular cross section of the file by rotary motion, and an uninstrumented area. As a result, root canal wall thickness after canal preparation becomes nonuniform, and a thin root canal wall caused by excess shaping may lead to root fracture and perforation post-preparation. Ni–Ti rotary instruments are effective for shaping of the narrow area along the root canal wall. However, the present results suggest that Ni–Ti rotary instruments are not suitable for C1 root canal morphology having a consecutive semi-circular slit that greatly opens from the root apex such as this study. In addition, the flexibility by the cross section design of the PTN and REC may have produced a difference in rotary shaping area to root canal walls.

SAF simultaneously shapes and cleans using a compressible open tube design and the outside irrigation function of the handpiece. The cylindrical lattice structure fits the cross section of the canal and shapes the circumference of the root canal uniformly, similar to filing by vertical vibration [32]. In SAF, the lingual side wall (groove portion) showed larger shaping area than the buccal side wall. This caused stronger contact with the file. The lingual side of the tooth structure may be thinner than the buccal side in the C-shaped root canal, so thinning of the lingual side root canal wall by excessive shaping may require attention. When the time taken for instrumentation in each group was compared, SSK needed approximately 6 min because of the multitude of instruments used, cumbersome technique and selective rotary motion. SAF needed about 4 min per root canal because shaping efficiency is inferior with vertical vibration, such as filing. In contrast, PTN and REC required less time (< 3 min) because they are simple techniques.

Hybrid instrumentation techniques combine the best features of different systems and instrumentation techniques [33]. The concept is to combine hand and Ni–Ti rotary instruments or different Ni–Ti rotary instruments. According to our findings, the combination of reaming and filing actions may achieve more effective and adequate instrumentation for complex systems such as C-shaped root canals. However, all groups showed differences in shaping area. According to Kuttler [34], the size of the apical foramen that varies according to a maturity degree of the root is #25 ~ #35 with mandibular molars. Thus, an uninstrumented area may be seen in root canal walls prepared with smaller files of around #25. In addition, Salzgeber and Brilliant [35] found that a minimum master apical file (MAF) size of #30 K-file allowed penetration of irrigants to the apex. In addition, the steps such as access cavity and straight line access omitted in this study are indispensable on the adequate root canal preparation in clinical practice. If these steps were neglected, it may contribute to the risk of inadequate instrumentation and file fractures because of the inappropriate insertion of instruments in root canals.

This study suggests the importance of correctly grasping the root canal system and features of instrument for optimal endodontic treatment. We recommend further studies including other instrument designs and working motion such as Ni–Ti rotary instruments of high stiffness and cutting capacity.
